# Bayesian non-negative factor analysis for reconstructing transcription factor mediated regulatory networks

**DOI:** 10.1186/1477-5956-9-S1-S9

**Published:** 2011-10-14

**Authors:** Jia Meng, Jianqiu (Michelle) Zhang, Yidong Chen, Yufei Huang

**Affiliations:** 1 Department of Electrical and Computer Engineering, University of Texas at San Antonio, San Antonio, Texas, USA; 2 Department of Epidemiology and Biostatistics, UT Health Science Center at San Antonio, San Antonio, Texas, USA; 3 Greehey Children’s Cancer Research Institute, UT Health Science Center at San Antonio, San Antonio, Texas, USA

## Abstract

**Background:**

Transcriptional regulation by transcription factor (TF) controls the time and abundance of mRNA transcription. Due to the limitation of current proteomics technologies, large scale measurements of protein level activities of TFs is usually infeasible, making computational reconstruction of transcriptional regulatory network a difficult task.

**Results:**

We proposed here a novel Bayesian non-negative factor model for TF mediated regulatory networks. Particularly, the non-negative TF activities and sample clustering effect are modeled as the factors from a Dirichlet process mixture of rectified Gaussian distributions, and the sparse regulatory coefficients are modeled as the loadings from a sparse distribution that constrains its sparsity using knowledge from database; meantime, a Gibbs sampling solution was developed to infer the underlying network structure and the unknown TF activities simultaneously. The developed approach has been applied to simulated system and breast cancer gene expression data. Result shows that, the proposed method was able to systematically uncover TF mediated transcriptional regulatory network structure, the regulatory coefficients, the TF protein level activities and the sample clustering effect. The regulation target prediction result is highly coordinated with the prior knowledge, and sample clustering result shows superior performance over previous molecular based clustering method.

**Conclusions:**

The results demonstrated the validity and effectiveness of the proposed approach in reconstructing transcriptional networks mediated by TFs through simulated systems and real data.

## Background

Transcription factor is one major gene regulator that governs the response of cells to changing endogenous or exogenous conditions [1]. Understanding how transcriptional regulatory networks (TRNs) induce cellular states and eventually define the phenotypes represents a major challenge facing systems biologists. So far, numerous models have been proposed to infer the transcriptional regulations by TFs including, ordinary differential equations, (probabilistic) Boolean networks, Bayesian networks, and information theory and association models, etc [2]. Ideally, the TF protein level activities are needed for exact modeling; however, due to low protein coverage and poor quantification accuracy of high throughput proteomics technologies such as protein array and liquid chromatography-mass spectrometry (LC-MS), the measurements of TF protein activities are currently hardly available. As a compromise, most of the aforementioned models conveniently yet inappropriately assume the TF’s mRNA expression as its protein activity. Given the fact that gene mRNA expression and its protein abundance are poorly correlated [3,4], these models cannot accurately model the transcriptional *cis*-regulation or reveal at the best TF *trans*-regulation.

In contrast, works based on factor models [5-10] point to a natural and promising direction for modeling the TF mediated regulations, where the microarray gene expression is modeled as a linear combination of unknown TF activities, and the loading matrix in this model indicates the strength and the type (up- or down- regulation) of regulation. However, due to distinct features of TF regulation, conventional FA model is not readily applicable. First, due to various reasons (normal and disease, cancer grade, subtypes, etc), the samples are usually not independent with each other but show some clustering effect; while in the existing FA models, factors are typically assumed independent, which, although true in many applications, is not a realistic assumption for TF medicated regulation. Secondly, since a TF only regulates a small subset of genes, the loading matrix should be sparse. While with constructions of TF regulation databases, such as TRANSFAC [11], the knowledge of TF regulated genes becomes increasingly available, and should be included in the model so as to boost signal-to-noise and improve performance [12]. The inclusion of prior information and sparsity constraint naturally call for a Bayesian solution. As an added advantage, having this prior knowledge actually resolves the factor order ambiguity of the conventional factor analysis. Thirdly, as suggested in [13-15], the non-negative assumption on TF activities be imposed.

In a response to these requirements of modeling TF mediated regulatory networks, we propose here a novel Bayesian non-negative factor model (BNFM). Different from conventional factor analysis models, BNFM consists of a sparse loading matrix and a set of correlated non-negative factors. The sparsity of the loading matrix is constrained by a sparse prior [16] that directly reflects our existing knowledge of TF regulation. That is if a gene is known to be regulated by a TF, then the prior probability that this regulation exists is high, and otherwise, very low due to the generic sparse nature of TF regulation (A TF only regulates a small number of genes in the whole genome). Because of clustering effect on the data samples, the factors in this BNFM model are considered to be correlated and modeled by a Dirichlet process mixture (DPM) prior [17]. DPM imposes a natural non-parametric clustering effect [18] among samples of the same TF and can automatically determine the optimal number of clusters. Moreover, since the activities of TFs are non-negative, they are assumed to follow a (non-negative) rectified Gaussian distribution [19]. Due to the complex nonlinear structure of the BNFM, the estimation of the model becomes analytically infeasible and highly complicated numerically. A Gibbs sampling solution is developed to infer all the relevant unknown variables.

## Method

### Bayesian non-negative factor model

Let  represent the *n*-th microarray mRNA expression profile of *G* genes under a specific context. In practice, microarray data **y***_n_* register the log2 scaled (fold change of) gene expression levels under the context of interest relative to a background often obtained as the average expression levels among a variety of contexts, such as different cell lines and tumors [20,21]. We assume that the expression level **y***_n_* is due to the linear combination of scaled TF absolute protein activites and modeled by the following factor model(1)

where,

**x***_n_*- the *n*-th sample vector of the scaled activities of *L* TFs of interest. Particulary, the non-negativity of **x***_n_* is modeled by applying the component-wise rectification (or cut) function *cut* to a vector pseudo factors **s***_n_*, such that the *l*-th element of **x***_n_* is expressed as(2)

Since clustering effects may exist among samples, the samples should be correlated. Therefore, pseudo factors **s***_n_* are modeled by a Dirichlet Process Mixture (DPM) of the Gaussian distributions as

where,  represents the Gaussian distribution with mean *µ_l_*_,_*_n_* and variance , *DP* denotes the Dirichlet process, and *NIG* is short for the conjugate Normal-Inverse-Gamma (NIG) distribution. This DPM model implies a clustering effect on **s***_n_* such that(3)

and(4)

where, *γ_n_* ∈ ℤ represents the cluster label of the *n*-th sample and is governed by a discrete GEM distribution [17], which defines the stick breaking process with parameter *α;* this implies that the elements of **s***_n_* are correlated. Based on (2) and (3), we have(5)

where,  denotes the rectified Gaussian distribution [19]. Since  and *γ_n_* are still defined in (4) by the DP, **x***_n_* is hence modeled by the DPM of the rectified Gaussian distributions and the elements of **x***_n_* are accordingly correlated. In contrast to the conventional mixture model, the DPM model enables the number of clusters to be learnt adaptively from the data instead of being predefined.

**A**- the *G* × *L* loading matrix, whose element *a_g_*_,_*_l_* represents the regulatory coefficient of the *g*-th gene by the *l*-th TF. Since a TF is known to regulate only small set of genes, A should be sparse. In our model, the elements of A are assumed to be independent and with the *a priori* distribution [16](6)

where, *π_g_*_,_*_l_* is the *a priori* probability of *a_g_*_,_*_l_* to be nonzero. For instance, if a TF regulates a total of 500 genes among the 20000 genes in the human genome, then *π_g_*_,_*_l_* is equal to

In most cases, *π_g_*_,_*_l_* are likely to be smaller than 10%. In practice, databases such as TRANSFAC [11] and DBD [22] provide information of experimentally validated or predicted target genes of TFs, and this knowledge can be incorporated in the model by setting, for instance, *π_g_*_,_*_l_* = 0*.*9, if TF *l* is known to regulate gene *g;* or otherwise *π_g_*_,_*_l_* = 0*.*025. The variable  defines how much the target genes are *loaded* on the corresponding TF and with prior distribution .

**c**- a vector of constant, which can be considered as the constant term retained when linearizing the general relationship **y***_n_* = *f*(**x***_n_*) as **y***_n_* = **Ax***_n_* + c. It may also be interpreted as static response of gene transcriptional expressions.

**e***_n_*- the *G* × 1 white Gaussian noise vector characterized by the covariance matrix  and with prior distribution .

**Figure 1:**
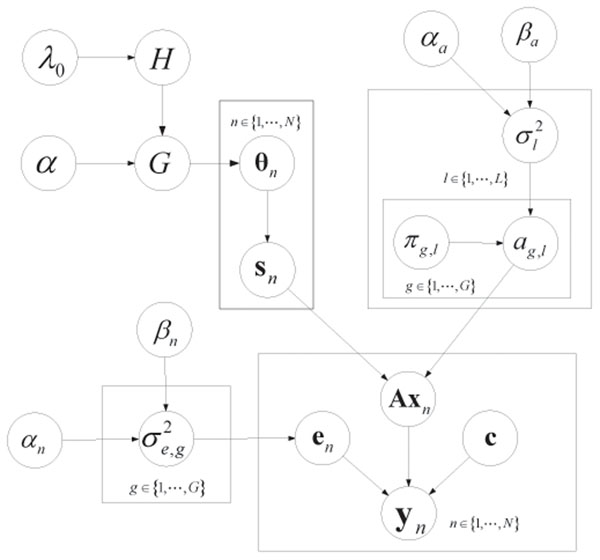
**Graphical model.** The Bayesian graphical model is shown here. **y***_n_* is the observed mRNA gene microarray data, the prior probability of regulation *π_g_*_,_*_l_* is extracted from TRANSFAC database, *α_n_*, *β_n_*, *λ*_0_, *α*, *αa*, *β_a_* are the hyperparameters, and the rest variables are unknown and need to be estimated.

The overall graphical model is shown in Fig.1.

### Equivalent model for centralized observations

To infer a factor model (1) more efficiently, the observation mean is usually removed at the first stage to eliminate the effect of the constant term c, resulting the equivalent model for centralized observations **ŷ***_n_*, where, **ŷ***_n_* = **y***_n_* – ***µ****_y_* and . Traditionally, since the models typically assume zero mean for the factors, the equivalent model for centralized observations remains the same except that the constant term is eliminated, i.e., if **y***_n_* = **Ax***_n_* + **c** + **e***_n_*, then, for the centralized data **ŷ***_n_*,(7)

and ***µ****_y_* can be viewed as an ML estimator of the constant term c [23]. For BNFM, however, since the factor mean is no longer zero, the equivalent model for BNFM no longer remains the same as above mentioned traditional model, but instead,(8)

where,(9)(10)

Given sufficient number of samples, the sample mean ***µ****_x_* = [*µ*_*x*_1__, *µ*_*x*_2__, …,*µ*_*x*_L__]^⊺^ can be approximated with the mean of prior distribution (4)(5), which can be calculated numerically. We can also see that the corresponding centralized factors are a shifted version of the original factors, and different samples shift the same amount, so sample clustering effect is still retained. On the other hand, the removed term from data centralization is no longer an estimator of the constant term **c**, but,(11)

The goal is to obtain the posterior distributions and hence the estimates of **A**, **x***_n_*∀*n*, *γ_n_*∀*n*, given **y***_n_*∀*n* and *π_g_*_,_*_l_* ∀*g*, *l*, which is the TF binding prior information extracted from existing database. For convenience, we let **Θ** denote all the known and unknown variables.

## Gibbs sampling solution

The proposed BNFM model is high dimensional and analytically intractable, so a Gibbs sampling solution is proposed. Gibbs sampling devises a Markov Chain Monte Carlo scheme to generate random samples of the unknowns from the desired but intractable posterior distributions and then approximate the (marginal) posterior distributions with these samples. The key of Gibbs sampling is to derive the conditional posterior distributions and then draw samples from them iteratively until convergence is reached. The proposed Gibbs sampler can be summarized as follows:

### Gibbs sampling for BNFA

Iterate the following steps and for the *t*-th iteration:

1. Sample  from ;

2. Sample  from ;

3. Sample  from *p*(*a_g_*_,_*_l_*|**Θ**_–*a*_*g*,*l*__);

4. for *n* = 1 to *N*

Sample  from ;

Sample  from ;

Sample  from *p*(**s***_n_*|**Θ**_–**s**_*n*__);

Note that  are marginalized and therefore does not need to be sampled. The algorithm iterates until the convergence of samples, which can be assessed by the scheme described in [24], [chap. 11.6]. The samples after convergence will be collected to approximate the marginal posterior distributions and the estimates of the unknowns. Since ***µ****_x_* can be approximated and calculated numerically, the factor **x***_n_* can be recovered from the centralized factor  with (9). The required conditional distributions of the above proposed Gibbs sampling solution are detailed in the next.

### Conditional distributions of the proposed Gibbs sampling solution

For simplicity, we let **x***_n_* and **y***_n_* denote the centralized factors and data in this section.

Let **E** = **Y** – **AX** and **e***_g_* = [*e_g_*_,1_, *⋯*, *e_g_*_,_*_N_*]^T^, then,

where, **ŷ***_g_*_,_*_l_* = **x***_l_a_g_*_,_*_l_* + **e***_g_*, **x***_l_* = [*x_l_*_,1_,*⋯*, *x_l_*_,_*_n_*]^T^ and **e***_g_* = [*e_g_*_,1_, *⋯*, *e_g_*_,_*_N_*]^T^*.* The posterior distribution of *a_g_*_,_*_l_*,(12)

where *Z*_0_ is a normalizing constant,  is the posterior probability of *a_g_*_,_*_l_* ≠ 0 and *BF*_01_ is the Bayes factor of model *a_g_*_,_*_l_* = 0 versus model *a_g_*_,_*_l_* ≠ 0

with  is the posterior distribution for *a_g_*_,_*_l_* ≠ 0 and defined by

where,  and , and *π_g_*_,_*_l_* is the prior knowledge of the probability of *a_g_*_,_*_l_* to be non-zero. When *π_g_*_,_*_l_* = 0*.*5, i.e, a noninformative prior on sparsity is assumed,  depends only on *BF*_01_, and  when *BF*_01_ > 1. Since model selection based *BF*_01_ favors *a_g_*_,_*_l_* = 0, it suggests that this Bayesian solution favors sparse model even when *π_g_*_,_*_l_* = 0*.*5.

It should be noted that *γ_n_* does not depend on **x***_n_* in the distribution. It is intended that samples of *γ_n_* from this distribution are not affected by the immediate sample of **x***_n_*, thus achieving faster convergence of the sample Markov chains. To derive this distribution, first let **ŷ***_l_*_,_*_n_* = **a***_l_x_l_*_,_*_n_* + **e***_n_* with **a***_l_* being the *l*-th column of **A** and hence . Then,(13)

where  denotes a new cluster other than the existing  represents the set of the pseudo factors besides *s_l_* that also belong to cluster *k*, *N*_–*l*,*k*_ is size of , and

with,

where,

and,

Noted that, for a new cluster,  and *N_–l_*_,_*_k_* = 0, and  can be derived from *g_k_* for  similarly.

This distribution can be expressed as(14)

where,  represents the truncated Gaussian with parameters  and between the interval (–*µ_x_*,+∞), and,

According to the graphical model, given *x_l_*,*n*, the conditional distribution of *s_l_*_,_*_n_* does not depend on **y***_1:N_*; As the predictive density *p*(*s_l_*_,_*_n_*|**s**_–_*_l_*_,_*_n_*, *γ_l_*) is shown to be a Student-t distribution, which can be conveniently approximated as a normal distribution when *N_–l_*_,_*_k_* is large:

and conditional distribution can be expressed as

where, *π*_*s*_*l*,*n*__ = 1 – sgn (*x_l_*_,_*_n_* + *µ_x_*)

Let the residuals **E** = **Y** – **AX**, and we have, , where **e***_g_* = [*e_g_*_,1_, *e_g_*_,2_, *…*, *e_g_*_,_*_N_*]^⊺^ Given the conjugate Inverse-Gamma prior, we have(15)

where Inv-Gamma represents the Inverse-Gamma distribution and

With the prior distribution , the conditional probability of  is,

where,  and , and *N_a_*_,_*_l_* is the size of .

## Results

### Test on simulated system

The proposed BNFM model was first tested on a simulated system, in which the microarray data consists of the expression profiles of 150 genes with 40 samples. The samples form 5 clusters and the 150 genes were assumed to be regulated by 10 TFs. The sparsity of loading matrix was set at 10%, which means that on average each gene is regulated by 1 TFs, and each TF regulates 15 genes. To simulate a practical imperfect database, the precision and recall of the prior knowledge were both set equal to 0.9 each, i,e., 90% of the database recorded regulations indeed happened in this specific data set (10% of the database recorded regulations may be context-specific and didn’t happen in the data); and 90% of the true regulations was recorded in the database (10% of true regulations are not in the database). This setting indicates that the recorded prior regulations may not exist in the experiment, and the unknown regulations could exist. Since this is a relatively large data set involving sampling of many variables, instead of examining convergence based on [24], [chap. 11.6], we adopted a more practical strategy by running a single MCMC chain for 10000 iterations with a burn-in period of 2000 iterations [25].

Since the algorithm estimates the loading matrix, the factors, the clustering result, and TF regulatory targets, to evaluate the performance, four respective metrics were computed. Particularly, in order to systematically evaluate the clustering result, a Van Rijsbergen’s *F* metric [26] that combines the BCubed precision and recall [27] was implemented as suggested in [28]. More specifically, let *L*(*e*) and *C*(*e*) be the category and the cluster of an item *e*. Then, the correctness of the relation between *e* and *e^′^* is defined by

That is, two items are correctly related when they share the same cluster. Moreover, the BCubed precision and recall are formally defined as

These two metrics can be further combined using Van Rijsbergen’s *F* metrics:

The *F* metrics satisfy all the 4 formal constraints defined in [28] including cluster homogeneity, cluster completeness, rag bag, and cluster size vs. quantity. We adopt the *F* metrics to evaluate the clustering result in the following tests. Similarly, a Van Rijsbergen’s *F* metric that combines the target prediction precision and recall is used to measure the target prediction result. Since our model can avoid sign ambiguity problem, the loading and factor estimations were evaluated using its Pearson’s correlation with their true values.

**Figure 2:**
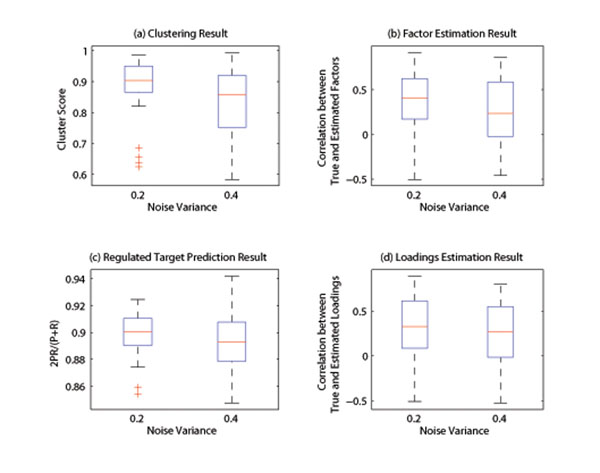
**Impact of noise.** The performance vs noise is shown here. Two noise conditions  are tested. It can be seen from the figure that, the algorithm performance deceases as noise increases. While the clustering result relies on noise heavily (Fig.2-a), the target prediction is relatively more robust against noise (Fig.2-c).

**Figure 3:**
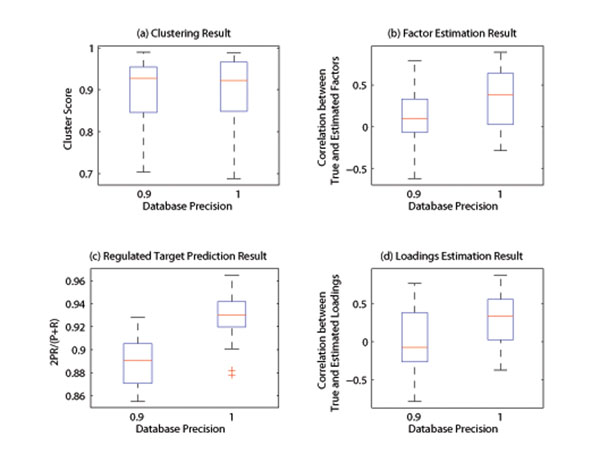
**Impact of database precision.** The performance vs database precision is shown here. It can be seen from the figure that, the algorithm performance deceases as noise increases. While the target prediction relies on the quality of database heavily (Fig.3-c), the clustering result is relatively robust against database quality (Fig.3-a) .

**Figure 4:**
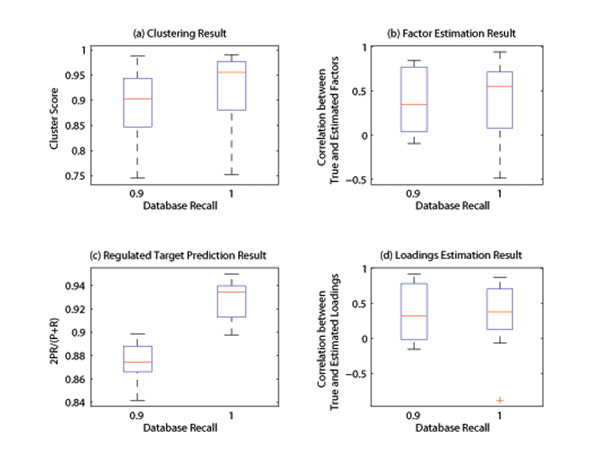
**Impact of database recall.** The performance vs database recall is shown here. It can be seen from the figure that, the algorithm performance deceases as noise increases. While the target prediction relies on the quality of database heavily (Fig.4-c), the clustering result is relatively robust against database quality (Fig.4-a) .

Experiments were carried out to test the impact of noise (Fig. 2), database precision (Fig. 3) and database recall (Fig. 4) on the performance of the algorithm. It can be seen from the simulation result that, at the low noise level or with high quality prior database, the developed algorithm can produce satisfactory result. Expectedly, the performance of the algorithm decreases as the noise variance increases or database quality decreases. However, the clustering performance is more sensitive to noise (Fig.2), while target prediction result relies more heavily on the quality of database prior knowledge (Fig. 3-4), because database directly support regulation posterior probability through its prior probability. In summary, the simulation results are indicative of satisfactory performance of the developed Gibbs sampling algorithm.

### Test on breast cancer data

**Figure 5:**
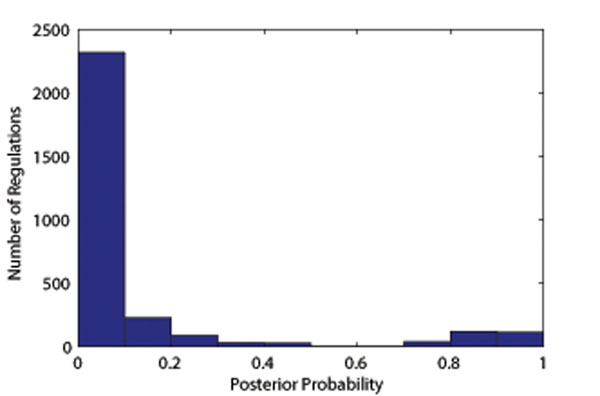
**Histogram of the regulation posterior probabilities.** The figure shows the histogram of posterior probabilities of all the possible regulations. Two distinct groups can be identified, each representing a group of regulations that are likely to happen (*p* > 0*.*5) or not (*p* < 0*.*5).

**Figure 6:**
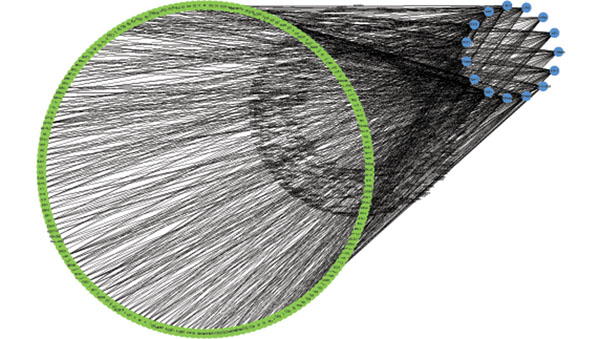
**Network of the posterior probability of regulations.** The network depicts possible regulations and their posterior probabilities (rounded by 0.1), where a edge indicates a possible regulation from a TF to a gene, and the line width of the edge represents the probability of the particular regulation exists, with thicker line width stands for larger probability; and vise versa.

After validating the performance of the proposed algorithm by simulation, the algorithm was then applied to the breast cancer microarray data published in [29-32]. Particularly, we applied the algorithm to 53 samples of grade 3 ER^+^ breast cancer. All samples came with gene microarray expression, ER status and survival time information. For the settings of the algorithm, we first manually selected a total of 15 TFs that are reported to be relevant to breast cancer (Table 1) and then retrieved a total of 199 regulated target genes (Table 2) by these TFs from TRANSFAC database [11] (Release 2009.4). We assume that TRANSFAC record has a 90% precision and 90% recall, suggesting that the known regulations may be context-specific and unknown regulations could exist. From the precision and the recall, the prior probability of the loading matrix can be determined. Based on these settings, the proposed approach was applied to the breast cancer data set to infer the underlying regulatory networks and TF activities. The posterior distribution of the loading matrix (Fig.5) gives insight into the sparsity of inferred TF mediated regulation. It can be seen that the posterior probability of regulations fall into 2 distinct groups, i.e., one group has very small posterior probabilities, which correspond to regulations that do not exist; while the other group have larger posterior probabilities, which correspond the regulations that are likely to exist. Fig. 6 depicts possible regulations and their posterior probabilities (rounded by 0.1) in a network, demonstrating the capability of the proposed approaches to identify possible TF regulated target genes.

**Table 1:** List of tested 15 TFs and aliases

	Name	Target	Aliases
1	EBP-*α*	21	BPc; C/EBP; C/EBP alpha; C/EBPalpha; CBP;
2	ETS-1	14	c-Ets-1; c-Ets-1 54; c-Ets-1A; Ets1; p54; p54c-Ets-1.
3	FOS	23	c-Fos; FBJ osteosarcoma oncogene; p55(c-fos).
4	MYC	11	c-Myc; MYC; v-myc myelocytomatosis viral oncogene homolog (avian).
5	CREB	22	ATF-47; CREB; CREB-341; CREB-A; CREB-isoform1; CREB1;
6	ATF-2	16	activating transcription factor 2; ATF2; CRE-BP1; CREB2; CREBP1;
7	EGR-1	24	AT225; early growth response protein 1;
8	EBP-*β*	23	AGP/EBP; ANF-2; C/EBP beta; C/EBP-beta; C/EBPbeta; CEBPB;
9	NF-*κ*B	28	NFkappaB; Nuclear Factor kappa B.
10	P53	20	ASp53; LFS1; NSp53; p53; p53as; RSp53; tp53; TRP53;
11	ATF-1	14	activating transcription factor 1; ATF1; EWS-ATF1; FUS/ATF-1;
12	STAT-3	12	acute-phase response factor; APRF;
13	STAT-1	19	signal transducer and activator of transcription 1.
14	AP-2	16	activating enhancer binding protein 2 alpha; activator protein-2;
15	CREB-1	19	ATF-47; CREB; CREB1; cyclic AMP response element-binding protein;

The tested 15 transcription factors and their aliases.

**Table 2:** List of tested 199 genes

	Symbol		Symbol		Symbol		Symbol
1	CXCR4	51	CD82	101	PENK	151	CDKN1A
2	CAT	52	HLA-DRA	102	PIM1	152	PTTG1
3	FOS	53	VIP	103	COL1A2	153	MITF
4	MT2A	54	INS	104	IL2RB	154	HBB
5	PSMB9	55	PTGS2	105	ZNF268	155	CSF1
6	DBH	56	APOA2	106	GSN	156	TIMP1
7	SERPINC1	57	FGFR2	107	TNFRSF10C	157	F9
8	CHEK1	58	CCND1	108	CXCL3	158	VHL
9	SCN3B	59	CASP1	109	CSNK2B	159	CD1A
10	F7	60	HBB	110	TRA@	160	SFN
11	ITGAX	61	COL2A1	111	HLA-DPB1	161	SOAT1
12	EIF4E	62	MDM2	112	TRA@	162	FCGR1A
13	TGFB2	63	RB1	113	TP53	163	FAS
14	CDC25A	64	NDRG1	114	SOX9	164	HBG1
15	IL3	65	BRCA1	115	ALOX5AP	165	WARS
16	SERPINE1	66	BAX	116	TOP1	166	KIR3DL1
17	IL10	67	ATF2	117	NFKB1	167	CD8A
18	F3	68	FN1	118	IL2	168	IL6
19	IL2RA	69	BCL2L1	119	SLC9A3	169	TWIST1
20	BDNF	70	CCR5	120	CYP3A4	170	CXCL1
21	WEE1	71	TF	121	CRH	171	IFNB1
22	CYP11A1	72	TFRC	122	CIITA	172	PTK2
23	NR4A2	73	HD	123	RFWD2	173	SPP1
24	VHL	74	CXCL1	124	LOR	174	CSF1
25	TRH	75	CSNK1A1	125	REN	175	TP73
26	SOD2	76	NR3C1	126	YBX1	176	CD53
27	CSF2RA	77	SPINK1	127	ATF3	177	NAB2
28	MUC1	78	EGR1	128	TEAD1	178	PTTG1
29	MEFV	79	EDN1	129	CDK4	179	IL1B
30	GNAI2	80	TFAP2A	130	APAF1	180	APOB
31	DRD1	81	CFTR	131	CYP19A1	181	IL8
32	ADRB2	82	MYC	132	ACE	182	TAF7
33	GCLC	83	FMR1	133	KRT16	183	PTP4A1
34	OPRM1	84	F8	134	NOS2A	184	HSD17B8
35	IFNG	85	TSC22D3	135	FXR2	185	ABCB1
36	BCL2A1	86	FGF2	136	IRF1	186	PBK
37	CCL5	87	LOR	137	CGA	187	TACR1
38	ICAM1	88	PTHLH	138	KRT14	188	MAOB
39	PSENEN	89	S100A9	139	ABCA2	189	RPL10
40	IER2	90	GADD45A	140	FGA	190	IVL
41	SOD1	91	EXO1	141	TALDO1	191	ERBB2
42	GNRHR	92	PLAU	142	CSF1	192	CCL2
43	LTA	93	PTH	143	SFTPD	193	BBC3
44	TERT	94	CDK4	144	CRP	194	TP63
45	TNFAIP6	95	PPARG	145	TPT1	195	RFWD2
46	ODC1	96	POLB	146	SLC9A2	196	FGFR4
47	LTF	97	ID1	147	CYP2A13	197	NAT1
48	PRLR	98	MT2A	148	DDX18	198	SELE
49	TNF	99	SST	149	CCNA2	199	FASLG
50	MMP1	100	KRT14	150	IL6ST		

The tested 199 genes.

**Figure 7:**
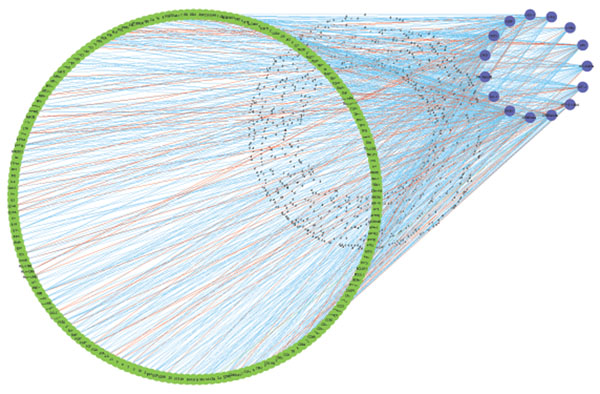
**Network of the regulation coefficients.** The network shows regulation coefficients, where an edge between a TF and a gene indicates the gene is regulated by the TF, and the color of the edge indicates the regulation types (red for up-regulation, and blue for down regulations), and the line width stands for the regulation strength.

When setting the cut-off threshold 0.5, the result confirmed 281 regulations among the 282 regulations that were defined in the TRANSFAC database, and identified 25 new regulations that are not recorded in the database. This fact demonstrates the ability of our algorithm to discover new regulations and discern context-dependent regulations among the prior knowledge, and the reconstructed network is shown in Fig. 7, showing the capability of the proposed approach to identify both the strength (represented by edge width) and the type (represented by edge color) of transcriptional regulations.

**Figure 8:**
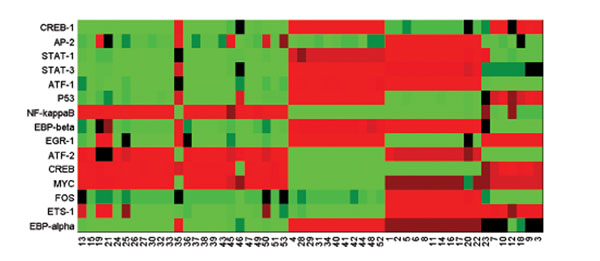
**Estimated transcription factor expression.** The tested samples fall into 3 major clusters with 24, 11 and 11 samples. The rest 7 samples may be considered as outliers that are not classified. In accordance with the sample clustering result, the recovered TF shows 3 major clustering patterns with a few outliers.

**Figure 9:**
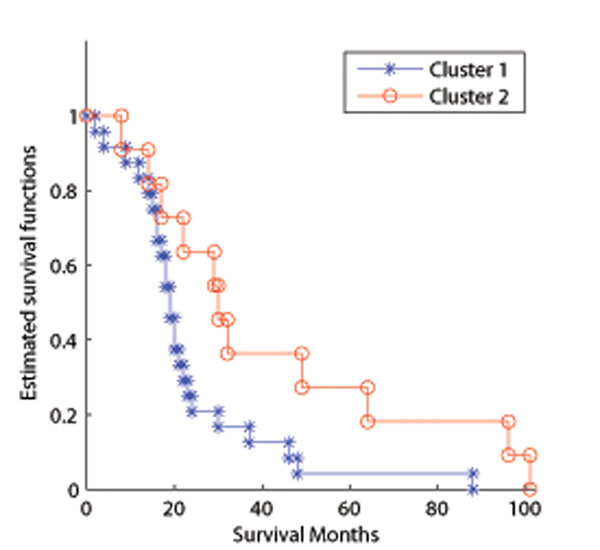
**Survival difference between cluster 1 and 2.** The survivals of the two estimated clusters show statistical difference *p* = 0*.*04 when using logrank test, indicating the two clusters are potentially corresponding to two subtypes of breast cancer that have different survival time.

Along with the estimates of regulatory coefficients, the transcription factor activities and the sample cluster attributes were also obtained. Fig.8 depicts the estimated TF activities, with the patient samples grouped according to the clustering result, and it clearly shows the coordinated clustering effects. To further gain insights into the clinical outcomes of different patient groups defined by the TF activities, survival analysis was carried out and it confirmed the survival difference between the the 1st and 2nd clusters (*p* = 0*.*05) as shown in Fig.9. Previous studies based on expression levels [33-36] identified 5 major subtypes (luminal A, luminal B, basal, ERBB2 overexpressing, and normal-like). We compared the pair-wise survival difference between our clustering (3 clusters) and previous result (5 clusters). It shows the superior performance of our method (Table 3) over the previous computation based method (Table 4).

**Table 3:** Survival test of clustering results

	Cluster 1	Cluster 2	Cluster 3
Cluster 1	N/A	0.04	0.16
Cluster 2	0.04	N/A	0.93
Cluster 3	0.16	0.93	N/A

The logrank test result of the survival difference between each pair of estimated clusters (min= 0.04). The survivals of cluster 1 and cluster 2 are significantly different with *p* = 0.04, indicating the two predicted clusters may each represent a subtype of breast cancer.

**Table 4:** Survival test of previous results

	luminal A	luminal B	Basal-like	HER2+/ER-	normal-like
luminal A	N/A	0.75	0.76	0.42	0.83
luminal B	0.75	N/A	0.98	0.7	0.8
Basal-like	0.76	0.98	N/A	0.67	0.94
HER2+/ER-	0.42	0.7	0.67	N/A	0.46
normal-like	0.83	0.8	0.94	0.46	N/A

The logrank test result of the survival difference between each pair of previous predicted clusters [33-36]. None of the pair shows statistical difference (min= 0.42).

### Discussion

We proposed a new approach to uncover the transcriptional regulatory networks from microarray gene expression profiles. We discuss next a few distinct features of it.

First, to reflect the fact that a TF only regulates a small number of genes among the whole genome, the loading matrix of the factor model is constrained by a sparse prior [16], which directly reflects our existing knowledge of the particular TF-gene regulation, i.e., if the regulation exists according to prior knowledge, the probability of the corresponding component of the loading matrix to be non-zero is large; or otherwise, very small. The introduction of sparsity significantly constrains the factor model and helps to enable the inference of a set of correlated samples.

**Figure 10:**
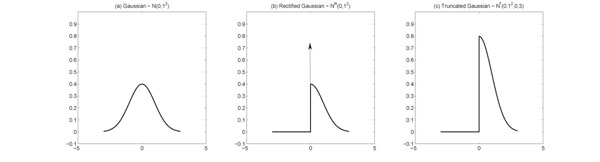
**Comparison of original, rectified and truncated Gaussian distributions.** The probability distribution function of Gaussian, rectified Gaussian and truncated Gaussian are shown in this figure. The range of Gaussian distribution  is from (–∞, ∞), the range of rectified Gaussian  is [0, *∞*], and the range of truncated Gaussian  is (0, 3).

Second, since the activities of TFs cannot be negative, the factors are modeled by a non-negative rectified Gaussian distribution [19], which not only is consistent with the physical fact of TF regulation but also avoids the inherent sign ambiguity problem of the factor models. Noted that, a rectified Gaussian distribution  is different from a truncated Gaussian  in that:

indicating that the rectified Gaussian model can also describe the possible suppressed state of TFs, which nevertheless cannot be modeled by the truncated Gaussian distribution. A comparison of Gaussian, rectified Gaussian, and truncated Gaussian is shown as Fig.10. In our model, the non-negativity is constrained only on the factor matrix; the elements of loading matrix can be either positive or negative, which models the corresponding up- or down-regulation of TFs. This is different from non-negative matrix factorization (NMF) [13,15,37,38]. NMF enforces that both the loading matrix and the factor matrix must be non-negative, i.e., all elements must be equal to or greater than zero. With the capability of modeling both the up- or down-regulations, the proposed BNFM is more appropriate for modeling the TF regulation than NMF.

To model the samples correlation due to, for instance, cancer subtypes, the samples are modeled by a Dirichlet process mixture (DPM), which imposes clustering effect among samples and can automatically determine the optimal number of clusters from data rather than be predefined in the algorithm. Forth, other types of data, such as ChIP-chip data [39-41] and DNA methylation data [42] can be conveniently integrated with gene expression data [43] under the proposed framework by setting a slightly different prior probabilities to the loading matrix. Integrating additional data types can potentially improve the accuracy of the reconstructed networks. [12].

However, the proposed model is not without shortcomings. First, this model can not yet capture regulations from TFs that are not specified in the prior knowledge database. In reality, it is possible that some TFs that are not specified in the prior knowledge actually regulate the gene transcription. Second, the algorithm may not converge in a reasonable number of iterations on a large data set, thus cannot yet be applied to genome wide data set. Because the model parameters are high dimensional and highly correlated, the speed of convergence may significantly slow down on a large data set [44,45]. However, such restriction on the size of genes and TFs forces us to focus the analysis on most relevant genes and TFs, making the analysis more targeted and easy to interpret. We demonstrate in section Result, how such analysis can be carried out starting from a whole genome microarray data. With the advancement in ChIP-seq technology and increasing knowledge of TFs biological functions, the proposed model could be applied for a genome-wide study in the future.

Thirdly, the prior knowledge may still need to be properly evaluated. If the prior knowledge is considered an estimation of the true TRN, when the precision *p*, recall *r* of prior information and the sparsity of the loading matrix *s* is given, the prior probability of the *g*-th gene to be a target of the *l*-th TF *π_g_*_,_*_l_* can be calculated as follows:

However, the precision or recall of the prior knowledge database are only arbitrarily specified (both 90%). In practice, the quality of prior knowledge should be evaluated first before more reasonable prior probabilities of regulations can be assigned.

## Conclusions

A Bayesian factor model that has sparse loading matrix, non-negative factors, and correlated samples, was proposed to unveil the latent activities of transcription factors and their targeted genes from observed gene mRNA expression profiles. By naturally incorporating the prior knowledge of TF regulated genes, the sparsity constraint of the loading matrix, the non-negativity constraints of TF activities, the regulation coefficients and TF activities can be estimated. A Gibbs sampling solution was proposed and model inference. The effectiveness and validity of the model and the proposed Gibbs sampler were evaluated on simulated systems. The proposed method was applied to the breast cancer microarray data and a TF regulated network for breast cancer data was reconstructed. The inferred TF activities indicates 3 patients clusters, two of which possess significant differences in survival time after treatment. These results demonstrated that the BNFM provides a viable approach to reconstruct TF mediated regulatory networks and estimate TF activities from mRNA expression profiles. The BNFM will be an important tool for not only understanding the transcriptional regulation but also predicting the clinical outcomes of treatment.
